# Considering Residents’ Health and Well-Being in the Process of Social Housing Redevelopment: A Rapid Scoping Literature Review

**DOI:** 10.1007/s11524-024-00915-2

**Published:** 2024-09-13

**Authors:** Jinhee Kim, Jennifer Green, Erica McIntyre, Christopher Standen

**Affiliations:** 1 Cities Institute, University of New South Wales, Sydney, Australia; 2 Centre for Primary Health Care and Equity, University of New South Wales, Sydney, Australia; 3 Collective for Midwifery, Child and Family Health, Faculty of Health, University of Technology Sydney, Sydney, Australia; 4 Institute for Sustainable Futures, University of Technology Sydney, Sydney, Australia; 5 Research Institute for Innovative Solutions for Well-Being and Health (INSIGHT), Faculty of Health, University of Technology Sydney, Sydney, Australia; 6 Health Equity Research and Development Unit, Sydney Local Health District, Sydney, Australia

**Keywords:** Social housing, Redevelopment, Health equity, Rapid scoping review

## Abstract

Many aging mid-twentieth-century social housing developments worldwide are set to undergo major redevelopment, aiming to improve residents’ living conditions. Nevertheless, the associated processes, particularly the challenges of relocation during the demolition and reconstruction phase, can significantly disrupt communities and social networks. Understanding the multifaceted impacts of social housing redevelopment projects is crucial to inform planning, design, and consultation for these projects. This scoping literature review explores how residents’ health and well-being are considered in the process of social housing redevelopment. We identified eight studies through a search performed on EMBASE, PubMed, and Scopus databases, with an additional hand search of the bibliographies of selected studies. A thematic analysis was conducted to identify the health and well-being impacts of the different phases during redevelopment projects. The findings demonstrate that social housing redevelopment projects have varied impacts on residents’ health and well-being depending on the subgroup of residents and the contextual characteristics of the original social housing estate. While improved physical infrastructure provides opportunities for better health outcomes, the disruption and lack of control during the relocation process may cause significant adverse health impacts. Moreover, the different phases during the redevelopment process expose different subgroups to varying risks. Based on these findings, we recommend that social housing redevelopment initiatives prioritize engaging and empowering residents to have better control in decision-making throughout all phases of the redevelopment.

**Supplementary Information:**

The online version contains supplementary material available at 10.1007/s11524-024-00915-2.

## Introduction

In the context of Western industrialized, high-income countries, social housing has been a crucial mechanism to provide secure and affordable housing for low-income households. Recognizing housing as a pivotal social determinant of health, social housing policies have sought to address the housing needs of low-income communities, thus contributing significantly to the promotion of health equity [1]. Rigorous empirical evidence in the US strongly supports a health-protective effect for social housing residents compared to a waitlisted control group or housing choice vouchers [2, 3].

However, the aging infrastructure of some of these housing complexes and the critical issue of poverty concentration have raised concerns about the deteriorating living conditions within these dwellings [4, 5]. For example, previous research has highlighted a strong correlation between residing in social housing and facing complex physical and mental health challenges, often exacerbated by the poor quality of the housing infrastructure [6–10]. Improvements in housing infrastructure have demonstrated positive impacts on the health outcomes of the residents, underscoring the critical role of living environments in shaping overall well-being [10]. Thermal comfort, reduced dampness and mold, and enhanced access to community facilities, further add to the potential benefits of well-executed redevelopment programs on the overall health and well-being of the residents.

In response, governments have undertaken redevelopment programs involving both the complete demolition and reconstruction of social housing complexes and renovation programs aimed at improving and updating existing structures. Redevelopment initiatives, exemplified by the United States’ HOPE VI program [11], Scotland’s SHARP initiative [12], and the proposed Waterloo redevelopment project in Sydney [13], often include mixed-tenure housing in the new complexes, i.e., a mix of social and privately owned/rented housing. However, critics argue that some redevelopment decisions prioritize financial goals and urban renewal objectives over the genuine well-being and interests of existing and future social housing tenants [14, 15].

Moreover, the impacts on the health and well-being of residents in the context of social housing redevelopment are multifaceted [11, 16]. Social capital and community support in these social housing complexes play a pivotal role in promoting the holistic health of residents [17, 18]. The *process* of social housing redevelopment introduces significant disruptions for the affected residents, manifesting at various redevelopment phases, from announcement, planning, and consultation, to the challenges of relocation during the reconstruction phase and the uncertainties surrounding residents’ eventual return or non-return after the project’s completion [19]. Regrettably, the planning processes for these redevelopment initiatives often overlook the holistic health implications, with most available programs focusing primarily on providing financial subsidies or vouchers for securing private housing, and offering limited social support during the relocation period [20]. These programs sometimes consider the individual needs of households in finding housing that meet their needs such as childcare, health and medical and other community services.

This scoping review aims to assess the current state of scholarship concerning studies that examine how residents’ health and well-being are considered in the process of social housing redevelopment projects. Through a systematic analysis of the existing literature, the study seeks to identify the key pathways and critical phases within the redevelopment process that significantly affect the physical and mental well-being of the residents. Additionally, the study aims to (a) inform future research directions on this topic and (b) provide valuable recommendations based on the documented evidence to inform future social housing redevelopment initiatives about the importance of considering the health implications for the affected communities in the planning and implementation of projects.

## Methods

The process of conducting this scoping review was guided by the recommendations presented by Arksey and O’Malley [21] and the PRISMA-ScR guidelines [22].

### Eligibility Criteria

We included studies that explored the association between health and well-being outcomes of residents of social housing dwellings exposed to redevelopment. Such redevelopment projects involve demolition of the old buildings and reconstruction into new mixed-tenure dwellings. Redevelopment thus involves re-housing of existing residents—this can be dwellings within the same estate if the redevelopment occurs in stages, though often involves relocation to other neighborhoods. We excluded social housing upgrade projects, such as the renovation of existing social housing and complexes, re-housing as a result of closing and demolition of social housing estates, and relocation as part of a program to rehouse social housing residents to subsidized private rental homes in less impoverished and less violent communities.

We maintained a broad perspective in considering the health outcomes that encompass the wider social determinants of health and other physical, mental, and social health outcomes. In summary, the eligibility criteria were the following:

- Population: residents of social housing dwellings (all age groups)
- Intervention: the process of redevelopment of social housing dwellings/estates including the decision to redevelop, demolition of social housing dwellings, rehousing during demolition and reconstruction, relocation after reconstruction
- Context: government-led redevelopment decisions of social housing buildings are generally accompanied by the demolition of the old social housing complex, (involuntary) relocation of residents, and rebuilding of the social housing complex into mixed-tenure development.
- Outcome: health and well-being outcomes and their determinants

Additional criteria for inclusion were type of publication (*peer-reviewed journal articles*); publication date (*all years up to 2022*); language (*English*); type of study (*quantitative, qualitative, mixed-methods*); and high-income countries. Papers that did not fit into the topic, non-empirical studies, or non-English publications were excluded.

#### Information Sources and Search Strategy

To identify relevant publications, we performed searches in three multidisciplinary databases: EMBASE, PubMed, and Scopus. The search strategy was developed based on three main concepts (*social housing* AND *redevelopment* AND *health and well-being impacts*). Search terms were developed using both medical subject headings (MeSH) and free-text keywords according to the nuances of the electronic databases. A draft of the search strategy was created by JC, which was subsequently reviewed, revised, and confirmed by all authors. The final search terms for each database can be found in Supplementary data 1.

An additional hand search was conducted on specific housing projects that were repeatedly mentioned in the selected publications, namely: HOPE VI (Housing Opportunities for People Everywhere, US Department of Housing and Urban Development), RAD (Rental Assistance Demonstration, US Department of Housing and Urban Development), and SHARP (Scottish Health, Housing and Regeneration Project). We conducted a focused grey literature search using Google and Google Scholar to capture studies on these specific housing projects.

#### Selection of Sources of Evidence

We identified a total of 581 publications through our electronic database search and an additional 20 publications from the focused grey literature search. After removing duplicates, JC conducted title and abstract screening, resulting in 94 full-text publications that were assessed for eligibility and were confirmed by a second reviewer, CS or JK. Upon applying the inclusion and exclusion criteria, a total of eight publications were included in the analysis.

#### Data Extraction

Research meetings were conducted between JG and CS to develop the data charting table and determine the definitions of the data extraction elements. The data extraction elements included: article title, first author year of publication, journal, aim of the study, region, main results, conclusion, data collection and analysis methods, health impacts, theme, strengths and limitations, challenges and opportunities, population groups, study size and length. Data extraction was conducted by JG and JK.

#### Analysis and Synthesis

After completing data extraction, we conducted a thematic analysis to identify and categorize studies based on their main findings and health and well-being outcomes. Studies were grouped based on the health outcomes and pathways impacted by the social housing redevelopment phases as they emerged from the publications. We used a descriptive narrative approach to present the findings according to each theme of health impact. The synthesis of findings is presented in the results section of this scoping review.

## Results

### Document Flow Diagram

**Fig. 1:**
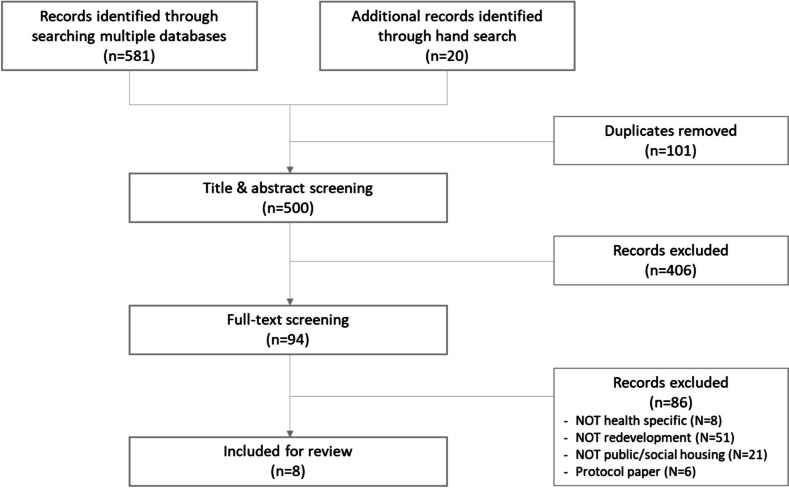
Document flow diagram

A total of eight studies were identified for inclusion in this literature review, as outlined in the PRISMA flowchart (Fig. 1).

### Study Characteristics

Out of the eight studies included in this scoping review, six were primary case studies, while two were reviews. Three of the six empirical studies were quasi-experimental studies using both quantitative and qualitative data in their analyses [20, 23, 24]. The other three were qualitative studies, examining the lived experience of tenants undergoing public housing redevelopment [25–28]. The publication years ranged between 2004 and 2020. All studies were conducted in high-income countries: USA (*n* = 6) and Australia (*n* = 2). All six studies from the USA were based on the federal HOPE VI public housing revitalization program.

Offsite relocation occurred during social housing redevelopment in most studies except in the study by Hagan et al. [28], where a one-for-one replacement of public housing units occurred and residents remained on site owing to a staged construction plan. Across the studies that address relocation, two distinct points of relocation were identified: relocation out of original social housing estates during the demolition and reconstruction period, and the subsequent return to the newly constructed social housing. Most of these studies examine the impacts of relocation during the demolition and reconstruction phase with a focus on comparing the impact on residents’ health and well-being based on their choice of relocation, i.e., relocating to subsidized private housing or other social housing. Only two studies specifically address the impact of returning to the redeveloped housing [20, 28]. A summary of the eight studies included in the review is presented in Table 1.

**Table 1:** Summary of publications included in the scoping review (*n* = 8)

First author, publication year	Title of article	Region	Aim of study	Characteristics of the social housing redevelopment project	Study characteristics	Key findings
*Empirical studies*						
Brooks, 2005 [23]	Resident perceptions of housing, neighborhood, and economic conditions after relocation from public housing undergoing HOPE VI redevelopment	Atlanta, USA	To examine residents’ perception of well-being in the areas of housing, neighborhood conditions, finances, and overall living situation after relocation	In 1999, residents were relocated to either private housing using vouchers or to other public housing estates	Mixed-method, post-test only design with two data points at 4 and 5 years after relocation Focus group interviews with 93 residents	Voucher users believed their house, neighborhood, and overall global living situation had improved since relocation. Moving was associated with residents perceiving their situations improving in many categories
Brooks, 2012 [20]	Voucher users and revitalized public housing residents 6 years after displacement	Atlanta, USA	To compare residents who returned to the redeveloped community and those who decided to keep living in subsidized private-sector housing	Demolished in 2000 and redevelopment completed in 2005. Two hundred sixty of the 520 new units were reserved for public housing residents. The original tenants had the option of keeping their housing vouchers, moving back to redeveloped public housing, or remaining in other public housing projects they had moved into	Mixed methods observational and descriptive study using quantitative and qualitative data collection Focus group interviews with 56 respondents	Residents who moved back to the revitalized public housing were highly satisfied with their housing, had significantly fewer material hardships, and perceived their economic well-being more positively compared to residents remaining in the voucher program
Webb, 2017 [24]	Finding HOPE: changes in depressive symptomology following relocation from distressed public housing	Charlotte, USA	To examine post-relocation depressive symptomology among relocated residents	During the relocation process, residents were paired with counselors, and tenants could relocate to either another public housing development or a private market housing with a voucher. Households also received Community and Supportive Services from contracted case managers	Pre/post-relocation survey (*n* = 127), several months before relocation and 1.5 years after relocation	Depressive symptomology substantially decreased following relocation, and those with higher CES-10 scores were more likely to move to other public housing rather than through the voucher program. Some residents were fearful of leaving public housing and assuming the responsibilities of a private-market rental unit
Hagan, 2020 [28]	Homeplace: care and resistance among public housing residents facing mixed-tenure redevelopment	San Francisco, USA	To explore the ways that residents of public housing negotiate, endure, and resist traumatic stress in their everyday lives and highlight the context and place-specific ways in which redevelopment may unsettle deeply rooted sociocultural configurations of home and community	Two long-standing, predominantly African American, public housing communities undergoing a public–private housing redevelopment initiative. Interviews were conducted during a 2-year period (2015–2016) while residents’ homes were being demolished and rebuilt	In-depth semi-structured interviews with 44 residents, constructivist grounded theory	Four subthemes constitute homeplace as a process: (a) homeplace emerging—shared knowing, caregiving, and lineage; (b) maintaining homeplace—networks of protection in unsafe contexts; (c) disrupting homeplace—consequences of redevelopment activities; and (b) reclaiming homeplace—community-envisioned ways of remembering, rebuilding, and preserving community
Crawford, 2017 [27]	Opportunity or loss? Health impacts of estate renewal and the relocation of public housing residents	Sydney, Australia	To investigate the potential health impacts of relocating residents as part of the redevelopment of a public housing estate in South Western Sydney, New South Wales	Since 2011, tenants have been relocated to enable demolition and construction work. The individual needs of affected tenants are considered by professional relocation officers. Tenants can relocate back to the estate once redevelopment works have been completed	In-depth, semi-structured interviews with 34 participants: 20 residents, 8 health staff, and 6 staff from local public housing authority	A range of current and potential health impacts, and satisfaction with the relocation process and outcomes, were linked to individual residents’ responses to the estate redevelopment and relocation, housing and neighborhood quality, residents’ social networks, and access to social and health care services. Health impacts differed for residents depending on a range of factors and a personalized approach was identified to contribute to positive health outcomes
Arthurson, 2016 [26]	Public housing renewal and social determinants of health	Melbourne, Australia	To examine aspects of the residential context that appeared important to the health and well-being of returning tenants	A three-stage project that started in 2006, with the first stage completed in 2011 at the time of the study	Both qualitative and quantitative methods: observations on the Estate and at community events, in-depth interviews with tenants, a survey of tenants, interviews with policymakers and community stakeholders, and interviews with private residents	Factors including the housing allocation process, safety and security, social networks, and green space were important to residents and were interpreted as being integral to their health and well-being
Abbott, 2019 [25]	Managing the hope and worry of housing renewal—supporting well-being in the emerging community	New South Wales, Australia	To examine challenges to promoting the well‐ being and social connectedness of social housing tenants during housing renewal	A social housing estate of 1470 single dwellings redeveloped to a low‐ to medium‐density housing area of approximately 2000 new homes in a 70% private to 30% public housing ratio	Qualitative interview data with nineteen people in community‐based work roles in housing, health, and social support agencies	Promoting access to services and supporting the mental health and social connectedness of residents are key goals; however, a lack of clarity on which services and community resources would exist in the new neighborhood remains a challenge to support residents
*Reviews*						
Keene, 2011 [16]	“Weathering” HOPE VI: the importance of evaluating the population health impact of public housing demolition and displacement	USA	To conceptualize different pathways and the potential contribution of relocation to weathering, a concept on the biophysical process to underlie early health deterioration and excess mortality, observed by African Americans	At most HOPE VI redevelopment sites, the vast majority of the original tenants are excluded from the criteria to return, leading to a large-scale relocation of low-income households and communities	Review of the HOPE VI literature, primarily relying on the HOPE VI Panel Study and the HOPE VI Resident Tracking Study. Additional data from in-depth interviews with relocated residents is utilized	Relocated HOPE VI residents have experienced few improvements to the living conditions and economic realities that are likely sources of stress and illness. Relocated residents must contend with these material realities, without the health-protective, community-based social resources that they often rely on in public housing
Popkin, 2004 [11]	The HOPE VI program: what about the residents?	USA	To study the impact of the transformation of public housing on the lives of its original residents and the barriers they face in making a successful transition	Focus on the relocation component of the multi-site HOPE VI revitalization program	Two systematic, multicity studies: the HOPE VI Panel Study (residents from five developments in 2001) and the HOPE VI Resident Tracking Study (former residents of eight properties in 2001, between 2 and 7 years after receiving the HOPE VI grant)	The effects of major dislocation are mixed, with some residents better off, others experiencing substantial hardship, and still others at risk of not being able to make a successful transition out of public housing

### Health Impacts of Social Housing Redevelopment Projects

The findings on the impact of social housing redevelopment projects on residents’ health and well-being derived from the eight publications included in this scoping review present a nuanced and varied landscape, influenced by the contextual characteristics of the original social housing development, the specific attributes of the redevelopment project and its processes, and the diverse needs and experiences of individual residents. Factors such as the physical and communal context of the original social housing developments, along with the features and location of the relocated housing, contribute to the complexity of these outcomes. Consequently, attempting to aggregate evidence for the purpose of generalization is conceptually unsuitable and methodologically challenging.

Considering the complexity of the literature, we emphasized the identification of recurring themes within the studies to construct a comprehensive narrative that highlights the impact of social housing redevelopment projects on residents’ health and well-being. Our presentation of the findings is structured based on the various phases of redevelopment, including the phases of announcement, planning, waiting to be rehoused, displacement, construction, and the eventual return to the redeveloped social housing. This framework was guided by the research questions and methodologies adopted by the included studies, allowing us to provide an in-depth understanding of how the various phases of the redevelopment process affect the overall well-being of the affected residents.

#### Lack of Control Over Redevelopment Decisions

Two publications examined the impact of residents’ participation in the redevelopment decision-making process. According to the findings of Crawford and Sainsbury [27], residents who actively engaged in the redevelopment activities expressed greater satisfaction than those who were less involved. The researchers linked being well-informed and empowered to participate with positive health outcomes, citing an increased sense of control and choice among participants. They also highlighted the effectiveness of a personalized approach aimed at providing information and minimizing residents’ stress and anxiety [27].

In the other study by Arthurson et al. [26], residents reported a lack of substantial public consultation regarding the redevelopment process and expressed dissatisfaction with the level of community consultation. The study highlighted a discrepancy between the residents’ expectations for housing allocation post-redevelopment and the actual allocations made. Residents voiced a lack of control over the decisions and that the department had failed to uphold its promises [26].

The lack of control over relocation decisions is associated with inevitable delays and uncertainties, leading to stress and anxiety among residents. These impacts were particularly pronounced among those with stronger attachment to their original communities, such as older residents and long-term occupants [27]. Moreover, residents were concerned about being overlooked and feared the possibility of being the last to be rehoused. The prospect of living in empty streets prone to crime and vandalism further amplified their stress and anxiety [27].

#### Relocation May Improve Material Benefits, Which is Offset by Relocation Stress and Social Disruption

Generally, the studies reported that the process of relocating from original social housing developments presents an opportunity for residents to enhance their living conditions, including improved indoor environment quality, access to green spaces, and better public transportation services. These physical environment upgrades offered notable health benefits, while the disruption of established social networks and communities can lead to adverse effects. These contrasting physical and social impacts were not only influenced by residents’ characteristics, such as age, length of tenure, and individual needs but also varied depending on the features of the new housing and neighborhoods.

#### Positive Health Impacts Through Improved Living Conditions

Social housing residents often face a range of physical and mental health risks, often exacerbated by the deteriorating conditions in their original housing. Consequently, moving to other housing and neighborhoods, typically in better condition, is generally associated with better health outcomes. Residents frequently reported elevated incidences of health issues like hypertension, asthma, type 2 diabetes, and depression, all of which are linked to factors such as poor indoor air quality, noise, pest infestations, and perceptions of crime and safety in their original housing estates [24, 27]. Post-relocation surveys reported improvements in satisfaction with housing environments and perception of safety, crime, and depression [24]. Additionally, some residents found that relocation offered an opportunity to break away from harmful relationships and situations, fostering a sense of responsibility and self-reliance and giving up unhealthy behaviors such as alcohol or drugs [23].

#### Health Benefits Offset by Disruption of Social Networks

The positive health and well-being impacts were offset by the disruption caused by uprooting [16]. The disintegration of communities, the loss of the concept of ‘homeplace’, and the reduction in access to concentrated social and medical services in the social housing estates can pose significant challenges. Residents who have stronger social support networks within their original neighborhoods showed more depressive symptoms post-relocation [24]. Even when relocation did not occur and the redevelopment was conducted in stages while residents still resided in their original estate, residents still felt a loss of homeplace during redevelopment and in rebuilding with disrupted social cohesion after redevelopment [28]. This situation is especially difficult for children [16] and older residents with complex needs who have deep roots in the community [27]. Individuals with multiple and complex needs present a more challenging housing situation, which makes the relocation process even more daunting [11]. Furthermore, relocation itself, even when undertaken voluntarily, is inherently stressful. However, when the decision to relocate is involuntary, as is often the case for most residents affected by social housing redevelopments, the challenges are compounded [27].

#### Health Impacts Differ Between Relocation Choice—Subsidized Private Housing or Other Social Housing

Most publications addressed residents’ health and well-being outcomes between the relocation to subsidized private housing or to other social housing. Programs aimed at supporting residents during the redevelopment phase typically form part of the overall redevelopment strategy, including financial assistance for relocation during the demolition and construction phases. Residents are often given the option of utilizing vouchers to secure subsidized private housing or opting for other social housing. Professional relocation counselors assist residents with finding suitable housing tailored to their needs. Two studies, in particular, examine the impact of relocation type and the influence on their health and well-being [23, 24].

#### Residents Who Relocated to Other Social Housing Have a Poorer Experience

It was consistently reported that people who relocated to other social housing estates generally experienced poorer health and well-being outcomes compared to those who moved to subsidized private housing [11, 20, 24]. These other social housing estates often persisted as distressed environments [27]. In some cases, the social housing estates to which they relocated would undergo subsequent redevelopment, resulting in further relocation.

Additionally, residents who moved into social housing tended to be older and often had pre-existing physical and mental health issues. Webb et al. [24] reported that residents suffering from depression opted for social housing over subsidized private housing. These individuals are particularly impacted by uncertainties and often require complex social and health services readily available in social housing complexes. The challenges associated with attending classes, passing credit checks, conducting house searches, and the fear of failing to meet requirements all serve as disincentives for residents to opt for private housing via voucher programs. This presents an extra burden to residents, given that residents with such complex needs may benefit significantly from the supportive environment provided by social housing complexes.

However, the experiences of residents also were associated with the specific characteristics of the social housing they relocated to. For instance, residents who moved into social housing projects located adjacent to a primary social hospital that treated low-income patients reported a high level of satisfaction compared to voucher users [23].

#### Residents Who Relocate to Subsidized Private Housing May Live in Better Environments But Suffer From Financial Stress

In contrast to residents relocated to social housing, people who moved to private housing using vouchers generally reported higher satisfaction and perceived improvements in various aspects of their living situation, including property condition, rent, utility bills, neighborhood safety, stress levels, and other related factors [23, 27]. This sentiment was particularly strong among younger residents, those with shorter tenures, and families with children. Voucher users also indicated better self-reported health compared to living in their original social housing homes, reflecting a more positive psychological outlook rather than actual improvement in physiological health [23, 27].

Relocating to mixed-tenure communities has been associated with improved access to public transport, recreational facilities, and supermarkets, thereby improving the overall quality of life and promoting healthy behaviors [27]. Living in such communities yielded positive health outcomes such as increased access to resources promotes healthy habits such as better nutrition and increased physical activity, simultaneously discouraging risky behaviors such as smoking, alcohol and substance abuse, violence, and crime [27].

The process of searching for a private home during relocation posed significant challenges. Difficulties often arose in finding rental properties that accept vouchers, leading to heightened levels of residential instability. The resulting financial strain was further compounded by escalating housing costs and increased utility bills (often three times higher than those in social housing), placing a disproportionate burden on residents’ income and impacting their ability to meet essential needs, such as food security [16, 23]. According to Brooks et al. [20], 74% of voucher residents reported that they were behind on their utility payments, compared to only 29% of social housing residents. Furthermore, perceived neighborhood safety tended to decline as social support was not as strong as in original social housing. As social support ties were disrupted, employment opportunities decreased due to fewer contacts and other job-seeking resources, and the absence of available childcare support from neighbors [16].

#### Returning to Redeveloped Housing Units

The redeveloped social housing estates often take the form of private, mixed-tenure developments, involving an application process for returning residents. It has been reported that only a small fraction of the original tenants return to the redeveloped sites [11]. Two studies included in our review specifically addressed the topic of residents returning to the redeveloped estates.

#### Most of the Returnees had Relocated to Other Social Housing During the Reconstruction

Brooks et al. [20] found only 8% returned to the redeveloped social housing, consistent with the US national average. Those who returned reported that they felt more comfortable in their original communities, and appreciated the physical improvements and safety of the newly constructed buildings [20]. This experience was particularly emphasized by residents who had relocated to distressed social housing during reconstruction.

In contrast, residents who had relocated to subsidized private housing during demolition and reconstruction were less likely to return. They considered vouchers a better commodity than redeveloped social housing, which acted as a disincentive for their return [20]. Moreover, the prospect of returning also entailed potential disruptions, leading some residents unwilling to uproot their families again. However, it is important to note that residents who continued to rely on vouchers still faced ongoing financial hardships in their subsidized private housing [20].

#### Moving into Redeveloped Housing is Generally Positive but with New Challenges

Returning to redeveloped sites generally yielded positive outcomes, although it introduced new challenges. Residents returning to redeveloped sites often experienced the benefits of increased green spaces and improved communal areas. However, their responses regarding safety and security varied, with some acknowledging the structural safety of the new buildings, enhanced by intercom systems and security cards, while others expressed concerns about the concentration of mental health issues, drug abuse problems, and disturbances caused by gatherings of young people, occasionally leading to intimidating situations [16, 26].

Despite efforts to minimize displacement and retain residents within the newly constructed community, individuals often expressed a profound sense of loss resulting from the disruption of their collective sense of place due to the design of the new development. The redeveloped sites also imposed regulations governing how residents can utilize the property, resulting in the loss of social space within the new structures [28].

### Policy Recommendations Mentioned in the Studies

In principle, redevelopment projects should enable individual tenants and residents’ associations to be equal players in planning, design, and activities [27], and minimize disruption to reduce stress and anxiety [27]. By doing so, these efforts will also enhance the collective well-being of the community [28].

Other recommendations offered were tailored more specifically to the phases of redevelopment. For example, in offering relocation options, it was suggested that full funding of housing vouchers is required especially in cases where other social housing options cause distress [23]. These financial assistance programs should align with rising utility and housing costs [20]. When relocating to other social housing units, authorities should make efforts to relocate residents to revitalized social housing projects [23].

Because residents with complex needs can become more vulnerable during rehousing, place-based services to meet these physical and mental health needs should be an integral part of the rehousing process [24, 27]. Support services need to include assistance to build social ties, gain employment, and acclimate to their new living environments [24]. Given the differing needs of different subgroups, a personalized approach should be considered at all phases [27]. For example, residents suffering from depression need extra support to access private-market housing, which is associated with better health outcomes [24]. Most importantly, supportive services should be comprehensive, and include effective case management [27].

## Discussion

This scoping review provides a narrative of health impacts that have been reported across the various phases of social housing redevelopment. The unexpectedly limited number of publications investigating the impact of the processes relating to social housing redevelopment projects on residents’ health and well-being, limited to just eight studies, highlights a significant gap in current research. Our review included only studies that examined the health and well-being impacts that follow through and are associated with the different processes involved in social housing redevelopment projects, while those that studied isolated aspects of relocation or displacement of low-income residents without the consideration of the broader context of social housing redevelopment projects were excluded. Consequently, despite a wealth of literature on the health and well-being impacts related to the outcomes of isolated dimensions of urban renewal, redevelopment, and gentrification, these studies fell outside the scope of this review [17, 29, 30]. Similarly, a substantial body of literature emphasized the improvement of housing and neighborhood conditions and its implications for the overall well-being of social housing residents. This emphasis indicates an existing gap in comprehensively examining the health and well-being impacts throughout the various phases of the social housing redevelopment process, including the announcement of redevelopment plans, consultation with the residents, demolition and relocation, and the subsequent return to newly constructed housing structures.

The findings on the impact on residents’ health and well-being were mixed and complex. Positive health and well-being impacts were observed from improved infrastructure, alongside negative impacts such as disruption of community and financial stress. These impacts were closely intertwined with residents’ contextual experiences, highlighting the intertwined dynamics of socio-cultural configurations within their homes and communities. Additionally, the impacts varied across different age groups, with older residents facing more pronounced challenges due to their deep-rooted connections with their original community, compared to younger residents who saw the relocation as an opportunity for change. This suggests the need for tailored interventions to address the diverse needs of these distinct resident groups during the redevelopment process [11]. Similarly, decisions regarding relocation or returning to social housing developments after redevelopment reflected these characteristics [11]. These findings suggest a risk of exacerbating disparities among populations already vulnerable to such challenges.

It is also important to note that the studies also report the distressed condition of the existing social housing complexes prior to redevelopment. The conditions to which the existing residents were exposed prior to upgrades were significantly poorer than comparable living conditions of nationwide social housing. Therefore, it is plausible that some improved outcomes reported by residents following redevelopment might be overinflated, due to the severely sub-optimal living conditions of the original social housing estates [11]. This observation highlights the need for governments and authorities to provide appropriate ongoing maintenance or renovation of social housing developments to ensure the health and well-being of their residents [31].

Moreover, the limited focus on the announcement and participation phases in planning presents an important area for future research. Understanding the contextual and differential impacts of social housing development projects across the different phases would be required to develop better policies and strategies to promote health equity and ensure the residents’ health and well-being needs are adequately met [32, 33]. Another area of further exploration includes investigations on residents’ health and well-being post-relocation. The findings of this review reveal this final phase of redevelopment projects is not addressed.

This rapid-scoping literature review has several limitations that warrant consideration. The broad definition of social housing redevelopment used in the search term across the literature may have led to the potential omission of relevant evidence despite careful consideration in the selection of search criteria. While three multidisciplinary databases were systematically searched, it is important to acknowledge the possibility of additional literature accessible through other database sources. Moreover, the absence of a quality assessment process during this review, combined with the considerable heterogeneity among the included studies, poses a risk of methodological bias. Furthermore, as the literature search was restricted to the English language, it is important to recognize that there may be pertinent evidence in other languages that was not included. Lastly, while the review process was a collaborative effort, the fact that only one reviewer conducted the selection, appraisal, extraction, and synthesis process implies a potential risk of bias.

## Conclusion

This review highlights the multifaceted and context-dependent nature of the health and well-being impacts associated with social housing redevelopment projects throughout the different phases. While the relocation to improved housing conditions and neighborhoods was generally associated with positive outcomes, the findings highlight the multifaceted challenges and complexities experienced by residents, including lack of control over redevelopment decisions, community disruption, and financial stress. The differential impacts across various resident groups, especially among older adults and residents with complex medical needs, call for tailored interventions to address the diverse needs of these populations in relocation and rehousing during construction. Furthermore, the review suggests social housing authorities invest in ongoing maintenance and renovation of social housing developments to ensure housing quality. Future research should focus on examining the differential impacts across redevelopment phases, with a focus on the residents’ participation and empowerment in the decision-making process.

## Supplementary Information

Below is the link to the electronic supplementary material.

Supplementary file1 Search terms. (DOCX 23 KB)

Supplementary file2 Data extraction sheet. (XLSX 28 KB)

## Data Availability

The data analyzed during the scoping are available as supplementary materials to this article.
