# Supplementary material for: Considering Residents’ Health and Well-Being in the Process of Social Housing Redevelopment: A Rapid Scoping Literature Review
Source: J Urban Health. 2024 Sep 13;101(5):1000–14. doi: 10.1007/s11524-024-00915-2 (PMC11461432; doi:10.1007/s11524-024-00915-2)
Supplement: Supplementary file 1 — Supplementary file1 Search terms. (DOCX 23 KB) [file 11524_2024_915_MOESM1_ESM.docx]

# Supplementary data 1. Search terms

## EMBASE

| Concept | SOCIAL HOUSING | REDEVELOPMENT | HEALTH AND WELL-BEING IMPACTS |
| --- | --- | --- | --- |
| Keywords, synonyms | **Embase:**  Exp housing/  Public housing.mp.  Affordable housing.mp.  Social housing.mp.  Low income housing.mp. | Exp city planning/  (includes urban renewal)  Urban redevelopment.mp.  Redevelop*.mp.  Renewal.mp.  Regeneration.mp.  Improvement.mp.  Rehousing.mp.  Change in tenure mix.mp.  Mixed tenure.mp.  Tenure mix.mp  Decant*.mp.  Displace*.mp. | **Embase:**  Exp health/  Exp Social determinants/  Exp wellbeing/  Exp health care/  Stress.mp  Anxiety.mp |

## PubMed

| Concept | SOCIAL HOUSING | REDEVELOPMENT | HEALTH AND WELL-BEING IMPACTS |
| --- | --- | --- | --- |
| Keywords, synonyms | Public housing [MeSH Terms]  Social housing [Text Word]  Affordable housing [Text Word]  Low income housing [Text Word] | Urban renewal [MeSH Terms]  Urban redevelopment [Text Word]  Redevelop* [Text Word]  Renewal [Text Word]  Regeneration [Text Word]  Improvement [Text Word]  Rehousing [Text Word]  Change in tenure mix [Text Word]  Mixed tenure [Text Word]  Decant* [Text Word]  Displace* [Text Word] | Health [MeSH Terms]  Social Determinants of Health [MeSH Terms]  Wellbeing [Text Word]  Well-being [Text Word]  Stress [Text Word]  Anxiety [Text Word] |

## SCOPUS

| Concept | SOCIAL HOUSING | REDEVELOPMENT | HEALTH AND WELL-BEING IMPACTS |
| --- | --- | --- | --- |
| Keywords, synonyms | “Public housing”  “Social housing”  “Affordable housing”  “Low income housing” | “Urban redevelopment”  “Urban renewal”  “Urban regeneration”  Redevelop*  Renewal  Regeneration  Improvement  Rehousing  “Change in tenure mix”  “Mixed tenure”  “Tenure mix”  Decant*  Displace* | Health  “Social determinants of health”  Healthcare  Wellbeing  “Well being”  Stress  Anxiety |
